# Factors determining satisfaction among facility-based maternity clients in Nepal

**DOI:** 10.1186/s12884-017-1532-0

**Published:** 2017-09-25

**Authors:** Suresh Mehata, Yuba Raj Paudel, Maureen Dariang, Krishna Kumar Aryal, Susan Paudel, Ranju Mehta, Stuart King, Sarah Barnett

**Affiliations:** 1 Nepal Health Sector Support Program, Ministry of Health and Population, Ramshaha Path, GPO Box: 7830, Kathmandu, 44600 Nepal; 2 Nepal Health Research Council, Ministry of Health and Population, Kathmandu, 44600 Nepal; 3 Institute of Medicine Tribhuvan University Maharajgunj, Kathmandu, 4600 Nepal; 4 Options Consultancy Services Limited, Devon House, 58 St Katharine’s Way, London, E1W1LB UK

**Keywords:** Quality of care, Client satisfaction, Maternity care, Utilisation, Nepal

## Abstract

**Background:**

With an increasing number of institutional deliveries, the Nepalese health system faces a challenge to ensure a quality of service provision. This paper aims to identify the determinants of client satisfaction with maternity care in Nepal using data from a nationally representative health facility survey.

**Methods:**

A total of 447 exit interviews, with women who had either recently delivered or who had experienced obstetric complications, were conducted across 13 districts in Nepal (87% in hospitals, 8% in Primary Health Care Centres (PHCCs), and 5% in Sub/Health Posts(S/HPs). Client satisfaction was measured using an eight item scale that covered accessibility, interpersonal communication, physical environment, technical aspect of care and decision making. A client satisfaction index was computed using ordinal principal component analysis. A multivariate probit model was used to assess the net effect of explanatory variables on client satisfaction.

**Results:**

Longer waiting times and overcrowding increased the likelihood of dissatisfaction. Having an opportunity to ask questions was positively associated with client satisfaction. Respondents from hill districts and rural areas were more likely to be satisfied in comparison to respondents from mountain, terai and urban areas. Socio-demographic factors (age, parity, caste/ethnicity, education, and ecological zone) and supply side factors (the time taken to reach a facility, type of facility, payment for services, and unknown heath worker or anyone entering the delivery room) were not statistically associated with satisfaction.

**Conclusions:**

The findings suggest client satisfaction with the quality of maternity services in Nepal could be improved by reducing waiting times and overcrowding, and giving the mothers adequate time to ask questions. If clients are more satisfied they are more likely to use the facility again/recommend to a friend.

## Backgroud

Satisfaction with maternity care is a multidimensional construct embracing satisfaction with self (personal control), and with the physical environment of delivery room and quality of care [1]. Aspects of care that may influence client satisfaction include: provider attitude, provider competence, outcome, physical environment, continuity of care, access, information, cost, bureaucracy and attention to psychosocial problems. Quality of care may not always be linearly associated with the level of satisfaction as perceived by the clients [2], however, client satisfaction is an important determinant of utilization of health services and the choice of health facility [3], it is important to know clients’ views and experiences while designing health programmes [4]. Client satisfaction also impacts the viability and sustainability of health care services, and is an important input for regular monitoring and quality improvement in health care delivery systems.

Hospital-based maternity care has contributed towards the reduction in maternal and neonatal mortality and morbidity in developing countries [5, 6]. With the increasing rates of institutional deliveries, poor countries are now facing a challenge to ensure good quality maternity care [7]. Access to and availability of institutional delivery care alone is not enough to achieve the desired goals in maternal and child health, it is also the quality of care that saves lives of mothers and newborns. Although client satisfaction is not directly addressed in the Nepal Health Policy 1991 [8], the Nepal Health Sector Programme-2 (NHSP-2: 2010–2015), includes client satisfaction as an essential component of health service delivery [9]. The newly released National Health Policy aims to develop an accountable health system that includes a mechanism to collect client complaints/feedback [10]. Several studies have described the determinants of clients’ satisfaction with maternity care in other countries [2, 11–14]. Women who are treated with respect, courtesy and dignity, and have trusting relationships with their care providers are more likely to be satisfied [15]. Lack of involvement in decision making and inadequate information about their care are associated with dissatisfaction [16]. Older people more commonly report satisfaction with maternity care [12–14, 17]. Compared to the service tracking survey 2012 (STS 2012), which showed 90% of clients satisfied/very satisfied) [18], STS 2013 showed a slightly lower satisfaction (86%) to maternity services [19]. Similarly, slightly higher percentage of maternity clients (4%) mentioned not willing to visit the facility again in STS 2013 [19] compared to STS 2012(3%) [18]. The two studies cited above (STS 2012 and STS 2013) have only looked into the percentage of clients satisfied but have not explored the determinants. This study will be helpful to address the knowledge gap regarding determinants of maternity clients satisfaction in the Nepalese context and similar settings.

## Methods

### Data source and sampling

This study is the secondary analysis of the survey data obtained as part STS 2013 conducted by the Ministry of Health (MoH), Nepal. The survey incorporated a facility questionnaire, and maternity and outpatient exit interviews. The primary objective of the survey was to provide national estimates for key reproductive, maternal, neonatal and child health indicators related to service availability, readiness and distribution of public (not private) sector of Nepal.

Nepal consists of 75 districts divided into three ecological zones (mountain, hill, and terai), or five administrative regions. For the purposes of the study design these ecological zones and administrative regions were amalgamated to form 13 sub-regions. The survey used a two-stage sampling design. In the first stage, one district was randomly selected from each of 13 sub-regions and these districts were the Primary Sampling Units (PSUs). This resulted in three districts being selected from the mountain region, five from the hill region, and five from the terai region, which are considered as strata for this study. In the second stage, the facilities were selected within each of the 13 districts. The higher the level of facility, the greater the probability of being selected: all public hospitals (17) and primary health care centres (PHCCs) (39) from the selected districts were included and an Equal Probability Sampling Method (EPSEM) was used to select health posts (HPs) (100/208) and sub-health posts (SHPs) (68/490). All maternity clients from PHCCs, HPs and SHPs attending the course of 2 days, and up to five clients per hospital, were interviewed. More details on the methodology are presented in the STS 2013 final report [20].

A total of 447 women who delivered at the facility or sought care for intra-partum complications were interviewed across the 13 districts (87% in hospitals, 8% in PHCCs, and 5% in S/HPs.). All clients who participated in exit interviews had received inpatient services. The smaller percentage of interviews at lower level facilities reflected the lower caseload.

### Data collection and quality assurance

Data collection was performed from July to August 2013. A total of 43 enumerators and 13 field supervisors were recruited for data collection. All of the field researchers had some experience of research/data collection, and had an academic background in health sciences (public health, nursing, medicine, sociology).

A Training Manual, Survey Field Manual, and Data Entry Manual were produced and used across the training period, data collection period, and data entry period, respectively to maintain quality and consistency in the work. Enumerators received 5 days of training. Supervision and monitoring visits from the Government of Nepal and Nepal Health Sector Support Programme (NHSSP) were carried out to identify and rectify any problems. During supervisory visits, some cases of non-compliance to survey manuals were observed. Feedback was provided during data collection and was also circulated to all of the district supervisors by telephone. Field teams could contact the central support team by mobile phone whenever necessary.

During fieldwork, all completed questionnaires were checked by the supervisors in the district before sending them to the central office for data entry. Feedback was provided to the enumerators during data collection.

### Data cleaning, coding and entry

Before data entry, consistency checks and content cleaning were carried out and outliers in continuous variables were checked. Any suspect data were cross-checked against hard copies of completed questionnaires. The database was developed in CSPro 5.0 and pre-tested before data entry started.

### Measures

#### Client satisfaction

Client satisfaction was measured by an eight-item instrument. The items covered key dimensions of client satisfaction: accessibility (one question), interpersonal communication (two questions), physical environment (two questions), clinical care (two questions) and decision making (one question). The responses were marked using a five-point Likert scale [21]: (1) very dissatisfied, (2) dissatisfied, (3) Neutral, (4) satisfied and (5) very satisfied. Different client satisfaction surveys have focused on different elements of client satisfaction. The eight items used in this survey aim to measure the key elements of the client’s experience of maternity care [22] and had a high internal consistency (Cronbach’s alpha = 0.74). Similar questionnaires were used in the previous rounds of the STS, conducted in 2011 and 2012, [18, 23] although some minor modifications were made to the 2013 STS questionnaire compared to earlier surveys.

The client satisfaction index was computed from the eight domains presented in Appendix, using the ordinal Principal Component Analysis (PCA) [24], since the variables were measured in the ordinal scale. A previous study has shown the agreement between observed satisfaction and predicted satisfaction using PCA [25]. A spearman correlations of the predicted overall satisfaction level was assessed with the observed satisfaction domains in this study and presented in Appendix.

To conduct bivariate and multivariate analysis the five point Likert scale was dichotomized: scores of four or five were counted as satisfied whereas one to three were counted as a not satisfied. The rationale behind the dichotomization was to yield a simple outcome variable which allows for complex tests of predictors.

### Explanatory variables

Individual characteristics (mother’s age, education and caste/ethnicity), cluster characteristics (ecological zone and place of residence), access to health facilities (reaching and waiting time), facility type, clinical care (mode of delivery and breastfed within an hour), advice and information (danger signs for mothers and newborns, exclusive breast feeding, comfort with the sex of the provider, family planning and being given the opportunity to ask questions), overcrowding within maternity departments, and the cost of care. Waiting time in this article refers to the duration of time between arriving at the facility and the first check-up by a service provider.

### Data analysis

Data analysis took into account the selection of districts within the strata defined by the ecological zones (mountain, hill and terai) and acknowledged the weighting of the data, the approximate stratification, and the clustering (health facilities asPSUs) while computing statistical tests, using the survey functions of STATA 12 SE Version.

To assess the determinants of clients’ satisfaction, the chi-square test was used for bivariate analysis and multivariate probit model was used to assess the net effect of explanatory variables on outcome variable. Findings are reported as significant where the *p*-value is <0.05 (significant at the 5% level).

## Results

More than two-thirds of the respondents were 20–29 years old (67%) and more than one-third were Brahmins/Chhetris (37%). Respondents with secondary education represented nearly half (46%) of the sample. It took less than 30 min for just over one-third of the respondents (37%) to reach to the health facility and most (82%) waited for less than 30 min before seeing a provider after reaching the facility. Half of the respondents perceived that the maternity department was overcrowded and more than four-fifths of mothers (83%) were breastfed within an hour. Almost two-thirds (65%) had paid for the service sought in the health facility. Half of the mothers were advised on the danger signs of mothers and newborns and less than two-thirds of mothers (61%) were advised on exclusive breastfeeding for 6 months (Table 1).

**Table 1:** Difference in level of satisfaction by socio-demographic, geography and supply side factors

Variables	Total		Satisfaction			
	N	%	Mean satisfaction score ^a^	Standard deviation	%^b^	*P*
Socio-demographic characteristics						
Age (years)						
< 20	109	24.4	3.16	1.55	50.0	0.712
20–29	300	67.1	3.21	1.46	47.5	
≥ 30	38	8.5	3.09	1.41	41.2	
Parity						
Primi	263	58.8	3.11	1.50	44.4	0.155
Multi	184	41.2	3.27	1.44	51.3	
Education status						
Never attended school	92	20.6	3.04	1.44	41.9	0.588
Primary education	57	12.8	3.21	1.40	45.2	
Secondary education	204	45.6	3.22	1.46	51.8	
Further education	94	21.0	3.26	1.59	46.3	
Caste/Ethnicity						
Brahmin/Chhetri	164	36.7	3.25	1.45	45.3	0.001
Terai/Madhesi other caste	91	20.4	2.94	1.53	38.3	
Dalits	68	15.2	3.15	1.49	50.0	
Newar	15	3.4	2.70	1.43	29.5	
Janajati	96	21.5	3.60	1.38	68.7	
Muslim	13	2.9	2.34	1.11	10.1	
Geographic/assessable factors						
Ecological zone						
Mountain	38	8.50	3.63	1.22	51.1	<0.001
Hill	136	30.4	3.70	1.27	68.5	
Terai	273	61.1	2.81	1.50	33.3	
Place of residence						
Urban	346	77.4	2.89	1.31	32.6	<0.001
Rural	101	22.6	3.25	1.12	80.6	
Reaching time (minutes)						
< 30	167	37.3	3.40	1.45	54.3	0.041
30–59	160	35.8	2.95	1.48	38.4	
≥ 60	120	26.9	3.15	1.46	48.5	
Supply side factors						
Type of health facility						
Higher level hospital	258	57.7	2.89	1.44	32.6	0.060
District hospital	129	28.9	3.07	1.45	52.6	
PHCC	38	8.5	4.38	0.93	83.2	
HP/SHP	22	4.9	3.96	1.39	73.2	
Waiting time (minutes)						
< 30	367	82.1	3.39	1.43	53.5	<0.001
30–59	28	6.3	2.00	1.27	19.3	
≥ 60	52	11.6	2.03	1.10	9.2	
Overcrowding at maternity department						
Yes	219	48.0	2.51	1.25	30.8	<0.001
No	228	51.0	3.13	1.35	63.8	
Paid for the service sought in health facility						
Yes	292	65.3	2.95	1.49	43.6	<0.001
No	155	34.7	3.56	1.36	53.6	
Interpersonal communication						
Give chance to ask any concerns						
Yes	117	26.2	3.69	1.40	66.1	<0.001
No	330	73.8	3.00	1.46	40.9	
Scolded by staff						
Yes	21	4.7	1.23	0.58	0.0	0.011
No	426	95.3	3.30	1.42	50.4	
Advised on danger signs of mother						
Yes	227	50.8	3.49	1.41	56.4	0.013
No	220	49.2	2.77	1.45	35.2	
Advised on danger signs of newborn						
Yes	218	48.8	3.52	1.39	56.8	0.012
No	229	51.2	2.77	1.46	35.7	
Advised for exclusive breastfeeding						
Yes	274	61.3	3.46	1.38	53.4	0.049
No	173	38.7	2.62	1.49	35.2	
Counsel on family planning						
Yes	168	37.6	3.58	1.32	59.6	0.511
No	279	62.4	2.89	1.51	38.5	
Structural factors						
Availability of drinking water						
Yes	240	53.7	3.45	1.39	56.7	0.029
No	179	40.0	2.66	1.47	30.4	
Don’t know	28	6.3	2.95	1.64	36.3	
Any unknown person entered in the delivery room						
Yes	64	14.3	3.27	1.45	33.9	0.213
No	365	81.7	2.97	1.45	50.1	
Don’t know	18	4.0	3.04	1.59	47.8	

^a^ the satisfaction score was constructed by giving scores: fully satisfied = 5, satisfied = 4, neutral = 3, dissatisfied = 2, fully dissatisfied = 1

^b^the satisfaction proportion was constructed adding the proportions: fully satisfied = 5; satisfied = 4

The mean satisfaction score and proportion of clients satisfied with maternity care is presented in Table 1. Satisfaction was highest among Brahmins/Chhetris compared to other caste/ethnic groups, and lowest among Muslims (Table 1). More than two-thirds of clients (69%) from the hill region and more than four-fifths (81%) from rural areas were satisfied, while only one-third of clients (33%) from urban areas and the terai were satisfied with the maternity care received. Clients who waited for less than 30 min before seeing a provider had higher satisfaction (54%) than those who had to wait for longer than 60 min (9%). Clients who reached the facilities within 30 min had higher satisfaction compared to those who travelled for more than 30 min. Clients who did not pay for the service and who did not report overcrowding at the health facility had higher satisfaction.

Overcrowding was more commonly reported at higher level hospitals (68%) than PHCCs and HPs/SHPs (2% and 0% respectively). It was also more common in urban (64%) compared to rural (14%) areas and in the terai districts (60%) compared to the mountain districts (4%). Furthermore, clients were more likely to have to pay for services at higher level hospitals and district hospitals (62% and 84% respectively) compared to HPs/SHPs (17%). A higher proportion of hospital deliveries was observed among Newars (98%) compared to other caste/ethnic groups, and the lowest proportion was observed among Muslims (68%) (data not shown).

Clients who received advice from health workers on danger signs for mothers and exclusive breastfeeding also had higher satisfaction. Clients who had an opportunity to ask questions to the health providers had higher satisfaction compared with those did not have that opportunity (Table 1).

Hence, bivariate analysis showed association of range of factors such as caste/ethnicity, ecological zone, place of residence (rural/urban), time taken to reach health facility, waiting time at health facility, overcrowding at health facility, payment for services, interpersonal communication skills of service provider and receipt of key informations (danger signs, new born care, exclusive breastfeeding) and structural factor (availability of drinking water) with maternity client satisfaction. However, multivariate analysis showed that those who had longer waiting times, i.e. at least 60 min, (satisfaction coefficient = −1.14, 95% CI (−1.77- -0.50) or between 30 and 59 min (coefficient = −1.00 95% CI (−1.61- -0.39)) and those who perceived the facility to be overcrowded (coefficient = −0.53,95% CI(−0.79- -0.28)) were more likely to be dissatisfied (Table 2). Residing in hill districts and rural areas, and having an opportunity to ask questions and breastfeeding within an hour of birth were positively associated with client satisfaction.

**Table 2:** Multivariate analysis

	Probit regression coefficient	95%CI		*p*
Age (years)				
< 20 (ref.)				
20–29	0.17	−0.33	0.67	0.504
≥ 30	−0.36	−1.06	0.34	0.320
Parity				
Primi (ref.)				
Multi	0.22	−0.07	0.51	0.143
Education status				
Never attended school (ref.)				
Primary education	0.09	−0.38	0.56	0.701
Secondary education	0.27	−0.06	0.59	0.108
Further education	−0.01	−0.45	0.42	0.954
Caste/Ethnicity				
Brahmin/Chhetri (ref.)				
Terai/Madhesi Other Caste	0.05	−0.31	0.40	0.792
Dalit	0.28	−0.11	0.67	0.161
Newar	−0.19	−0.90	0.52	0.594
Janajati	0.17	−0.43	0.77	0.588
Muslim	−0.69	−1.72	0.35	0.195
Ecological zone				
Mountain (ref.)				
Hill	0.89	0.41	1.37	<0.001
Terai	0.44	−0.15	1.02	0.141
Place of residence (rural = 1)	0.74	0.30	1.18	0.001
Reaching time (minutes)				
< 30 (ref.)				
30–59	0.08	−0.30	0.46	0.675
≥ 60	−0.03	−0.28	0.23	0.845
Type of health facility				
Higher level hospital (ref.)				
District hospital	0.14	−0.23	0.52	0.458
PHCC	−0.03	−0.75	0.69	0.931
HP/SHP	−0.37	−1.21	0.47	0.393
Waiting time (minutes)				
< 30 (ref.)				
30–59	−1.00	−1.61	−0.39	0.001
≥ 60	−1.14	−1.77	−0.50	<0.001
Overcrowd at maternity department	−0.53	−0.79	−0.28	<0.001
Paid for the service sought in health facility	−0.20	−0.62	0.22	0.357
Give chance to ask any concerns	0.70	0.33	1.06	<0.001
Advised on danger signs of mother	0.17	−0.26	0.60	0.436
Advised on danger signs of newborn	−0.04	−0.53	0.45	0.866
Advised for exclusive breastfeeding	0.06	−0.28	0.39	0.743
Counsel in family planning	0.11	−0.30	0.51	0.604
Availability of drinking water at facility				
Yes (ref.)				
No	−0.24	−0.64	0.16	0.233
Don’t Know	−0.30	−0.79	0.18	0.216
Any unknown person entered in the delivery room				
Yes (ref.)				
No	−0.07	−0.35	0.21	0.616
Don’t know	−0.03	−0.82	0.77	0.948
_cons	−1.20	−2.36	−0.03	0.044

*Note*: due to collinear relationship the variables clients were scolded by providers was excluded from this multivariate analysis

## Discussion

Client satisfaction with health care has been argued as a subjective as well as dynamic perception of the extent to which expected health care is received [7, 26, 27]. This study aimed to measure the determinants of client satisfaction. Specifically, the attribution of client characteristics, demand-side and supply-side factors on client satisfaction were investigated. The findings from the current study revealed that women wanted prompt attention without overcrowding. In addition, allowing mothers to ask questions was significantly associated with satisfaction with maternity care.

Factors such as ecological zone and place of residence were significantly associated with client satisfaction. Clients from rural areas were more satisfied with the care provided at the facility compared with clients from urban areas. Higher satisfaction among rural clients can be partly attributed to their lower educational and empowerment level and lower socio-economic class compared to urban counterparts. Women from rural areas might be less empowered to compare and express satisfaction compared with those from urban areas. Literature suggests that economically middle-class women prefer personal choice and less professional dominance during childbirth in comparison to lower-class women [28, 29]. Furthermore, Nepal’s health care delivery system is a mix of private and public health institutions. While there is a better choice in urban areas, only public health facilities are available in rural areas. Women from urban areas may be more familiar with the care provided by private health facilities. Literature suggests prior exposure and information from friends/relatives to be a determinant of women’s perception of maternity care [30].

Clients who were given enough time and attention to ask questions to better understand their care and their health status were more likely to be satisfied. Being able to address any concerns will give clients more confidence in the services provided, resulting in increased satisfaction. A study conducted in Sri Lanka found that clients who were provided with adequate information regarding newborn care, breastfeeding and others after examinations were found to be more satisfied [15] and another systematic review showed a strong correlation between satisfaction and education/information given [31].

This study revealed that longer waiting times increased the likelihood of dissatisfaction among clients, which is consistent with the findings of a study conducted in Bangladesh [2]. Prompt attention after reaching a facility has been found to be significantly associated with client satisfaction [32]. This may be due to higher opportunity costs associated with the visit, i.e. this time could be used for other income generating activities by family members. In addition, overcrowding at the facility is negatively associated with client satisfaction. This finding is consistent with other studies [33]. Overcrowding might affect numerous indicators of satisfaction and quality of care, including lead to longer waiting times, less time with providers, lack of bed space, and early discharge. A previous study from Nepal reported overcrowding, mainly at the higher level health facilities [34]. The findings suggest to a need for renewed focus on strengthening lower level maternity facilities and building the capacity of peripheral health workers to develop trust on them. Furthermore, a strong referral network need to be made functional with a provision of free transportation from a birthing centre to referral hospital [34].

Current study showed that Brahmin/Chettri clients had the highest satisfaction and Muslims the lowest. It is not clear whether this independent association reflects differences in clients’ expectations or reflects actual differences they had with the quality of care. Differences in client satisfaction by ethnicity have been noted in other settings as well [35].

The mean satisfaction score was higher for lower level facilities (PHCCs or below) than higher level facilities (district hospitals or above). However, the likelihood of satisfaction was not significantly different by level of health facility in the multivariate analysis. Previous studies have been inconsistent, with some showing clients attending higher level facilities to be more satisfied than those attending community level facilities [36], and others reporting clients were more satisfied with the lower level health facilities [15, 37, 38].

Client’s age did not have any significant association with client satisfaction, which is in contrast to another study which showed that age has a statistically significant effect on client satisfaction [14]. Older clients were more likely to be satisfied as demonstrated by studies conducted in Riyadh [17], northeast France [13] and the United States [12, 14] while some other studies have reported that age has a minor influence on client satisfaction [16, 39]. One of the explanations for age not being associated with satisfaction here could be a narrow age range of maternity clients which might have shaped similar expectations.

In contrast to the findings from studies conducted in Riyadh [17] and Kuwait [40] showing higher educational status of clients to be positively associated satisfaction. Studies conducted in South Africa [41] and India [36] showed greater dissatisfaction among higher educated parents. Similar to this study, a South African study found that satisfaction level did not vary much with clients’ socio-demographic characteristics [41].

Although the extent of disrespect and abuse in health facilities has not been systematically documented or well-defined in Nepal, a qualitative study revealed that disrespect and abuse in childbirth represents an important barrier to utilization of skilled birth care and other health care at health facilities [42]. In line with a this study [42], our study also reported significantly lower satisfaction among those who were scolded at facilities by the health providers. This finding suggests communication skills and behaviour improvement should be integrated with other health related trainings. This study revealed that payment to the services was not linked to client satisfaction; the perceived competence, assurance of treatment irrespective of ability to pay were the determining factors to trust in health care, may lead to higher satisfactions [11].

The findings are prone to some limitations. Since only a small number of respondents could be interviewed from Health posts and Sub health posts (5% of total respondents), due to low caseload, the findings may be more relevant to hospitals in Nepal. Since client satisfaction has been measured from client exit interviews, satisfaction is likely to have been over reported compared to data collection at the community level. It is debatable whether clients’ reporting of satisfaction truly reflects the quality of care received [26]. Previous studies highlight the gap between clients’ perception of high quality care and that perceived by health care professionals [2]. Client satisfaction was measured against the environment, however, studies have shown that satisfaction with self/personal control is also an important element of maternity client satisfaction [1]. Exit interview survey clients have some level of satisfaction with that particular facility and have made a choice to come there. This method of data collection may miss the views of those who are actually dissatisfied with that facility and have gone elsewhere. Likewise, providers may also perform better when they are aware that they are being observed or their clients are being interviewed. We assessed maternity client satisfaction based on background characteristics of clients and client perception to selected supply-side factors. However, there are other potential factors that may affect client satisfaction such as internal factors [43] and supply-side factors such as availability of drugs, mode of delivery, length of stay in health facility,contact-time with service providers, and others [22]. In addition, the primary aim of the STS survey was to compare service provision, distribution and client satisfaction across types of public health facilities and to assess progress over survey years rather than studying determinants of client satisfaction.

## Conclusions

This study did not find any differences in clients’ satisfaction with socio-demographic characteristics aside from caste/ethnicity. However, key supply side factors, such as longer waiting times and overcrowding at facility, were associated with poor client satisfaction, whereas getting an opportunity to ask questions was positively associated with client satisfaction, suggesting the quality at the point-of-care will be instrumental in order to enhance client satisfaction.

## Appendix

**Table 3:** Questionnaire for measuring client satisfaction

	Very unsatisfied	Unsatisfied	Neutral	Satisfied	Very satisfied
How satisfied were you about the waiting time?	1	2	3	4	5
How satisfied are you with the information you received from the providers?	1	2	3	4	5
How satisfied are you with the level of skill the provider had to deliver your baby?	1	2	3	4	5
How satisfied are you with the politeness of the staff with whom you consulted?	1	2	3	4	5
How satisfied are you regarding your involvement in decision makingduring the care at the facility?	1	2	3	4	5
How satisfied are you with the cleanliness of the facility?	1	2	3	4	5
How satisfied are you with the level of privacy you received?	1	2	3	4	5
How satisfied are you with the care you received at this facility?	1	2	3	4	5

**Table 4:** spearman correlation coefficients of predicted overall satisfaction with observed domains of satisfactions

	Spearman correlation coefficient	*P*
How satisfied were you about the waiting time?	0.446	<0.001
How satisfied are you with the information you received from the providers?	0.662	<0.001
How satisfied are you with the level of skill the provider had to deliver your baby?	0.589	<0.001
How satisfied are you with the politeness of the staff with whom you consulted?	0.620	<0.001
How satisfied are you regarding your involvement in decision making during the care at the facility?	0.567	<0.001
How satisfied are you with the cleanliness of the facility?	0.473	<0.001
How satisfied are you with the level of privacy you received?	0.541	<0.001
How satisfied are you with the care you received at this facility?	0.548	<0.001

## Abbreviations

CSPro: Census and Survey Processing System
EPSEM: Equal Probability Sampling Method
HP: Health Post
MOHP: Ministry of Health and Population
NHSP: Nepal Health Sector Program
NHSSP: Nepal Health Sector Support Program
PCA: Principle Component Analysis
PHCC: Primary Health Care Centre
PSU: Primary Sampling Unit
SHP: Sub-Health Post
STS: Service Tracking Survey
